# Shared Genetic Architecture and Causal Relationship Between Asthma and Cardiovascular Diseases: A Large-Scale Cross-Trait Analysis

**DOI:** 10.3389/fgene.2021.775591

**Published:** 2022-01-20

**Authors:** Yi Zhou, Zhi-Sheng Liang, Yinzi Jin, Jiayuan Ding, Tao Huang, Jason H. Moore, Zhi-Jie Zheng, Jie Huang

**Affiliations:** ^1^ Department of Global Health, School of Public Health, Peking University, Beijing, China; ^2^ College of Arts and Sciences, Boston University, Boston, MA, United States; ^3^ Department of Epidemiology and Biostatistics, School of Public Health, Peking University, Beijing, China; ^4^ Department of Biostatistics, Epidemiology and Informatics, Institute for Biomedical Informatics, University of Pennsylvania, Philadelphia, PA, United States; ^5^ Institute for Global Health and Development, Peking University, Beijing, China

**Keywords:** asthma, heart failure, shared genetics, genetic correlation, Mendelian randomization

## Abstract

**Background:** Accumulating evidence has suggested that there is a positive association between asthma and cardiovascular diseases (CVDs), implying a common architecture between them. However, the shared genetic architecture and causality of asthma and CVDs remain unclear.

**Methods:** Based on the genome-wide association study (GWAS) summary statistics of recently published studies, our study examined the genetic correlation, shared genetic variants, and causal relationship between asthma (*N* = 127,669) and CVDs (*N* = 86,995–521,612). Statistical methods included high-definition likelihood (HDL), cross-trait meta-analyses of large-scale GWAS, transcriptome-wide association studies (TWAS), and Mendelian randomization (MR).

**Results:** First, we observed a significant genetic correlation between asthma and heart failure (HF) (Rg = 0.278, *P* = 5 × 10^−4^). Through cross-trait analyses, we identified a total of 145 shared loci between asthma and HF. Fifteen novel loci were not previously reported for association with either asthma or HF. Second, we mapped these 145 loci to a total of 99 genes whose expressions are enriched in a broad spectrum of tissues, including the seminal vesicle, tonsil, appendix, spleen, skin, lymph nodes, breast, cervix and uterus, skeletal muscle, small intestine, lung, prostate, cardiac muscle, and liver. TWAS analysis identified five significant genes shared between asthma and HF in tissues from the hemic and immune system, digestive system, integumentary system, and nervous system. *GSDMA*, *GSDMB*, and *ORMDL3* are statistically independent genetic effects from all shared TWAS genes between asthma and HF. Third, through MR analysis, genetic liability to asthma was significantly associated with heart failure at the Bonferroni-corrected significance level. The odds ratio (OR) is 1.07 [95% confidence interval (CI): 1.03–1.12; *p* = 1.31 × 10^−3^] per one-unit increase in log_e_ odds of asthma.

**Conclusion:** These findings provide strong evidence of genetic correlations and causal relationship between asthma and HF, suggesting a shared genetic architecture for these two diseases.

## Introduction

Asthma is a heterogeneous disease with a strong genetic basis and is a major global public health problem worldwide due to its high prevalence and inadequate disease control (Barnes, 2011; Yang et al., 2017). Cardiovascular diseases (CVDs) including heart failure (HF) are a rapidly growing public health threat globally (Roth et al., 2017) and confer a substantial burden to the healthcare system (Ziaeian and Fonarow, 2016). Globally, both asthma and CVDs have increased over the past several decades, measured by disease incidence rate (for asthma) or prevalence (for CVDs) (Gottdiener, 2017).

Previous studies have reported overall convincing positive associations between asthma and CVDs (Tattersall et al., 2016; Sun et al., 2017). Observational studies have found that a history of asthma was associated with higher adulthood left ventricular mass index (LVMI) and hypertension (Sun et al., 2017). A large-cohort study of adults found late-onset asthmatics had an increased risk of incident CVDs that persisted after adjustment for age, sex, and CVD risk factors (Tattersall et al., 2016). In previous large multi-ethnic cohort studies, persistent asthmatics had a higher CVD event rate than non-asthmatics (Tattersall et al., 2015; Tattersall et al., 2020; Wee et al., 2021) and adult patients with asthma had increased risks of HF incident (Iribarren et al., 2012). Patients with asthma–chronic obstructive pulmonary disease (COPD) overlap syndrome (ACOS) were associated with a higher risk of HF (Yeh et al., 2017). It has been well known that observational studies are susceptible to reverse causality and confounding; therefore, whether asthma plays a causal role in the development of CVDs or vice versa remains largely unknown (Gottdiener, 2017). In particular, inflammation is involved in the pathophysiology of both diseases and therefore becomes a key confounder to study the causal effect between these two traits (Gottdiener et al., 2000; Fahy, 2015). Genetic studies showed that genetic markers of inflammation play important roles in the development of both CVDs and asthma (Raman et al., 2013), implying a common genetic architecture between asthma and CVDs. Besides the investigation of inflammation and potentially a few other similar focused studies, to our knowledge, no large-scale, genome-wide study has systematically reported the shared genetic loci between asthma and CVDs.

In this study, we conducted genome-wide, large-scale, cross-trait analyses to examine the genetic correlation and identify shared variants between asthma and CVDs. These included genome-wide genetic correlation analyses, Mendelian randomization (MR)-based causal inference, and functional enrichments analyses.

## Methods

### Genome-Wide Cross-Trait Analysis Study Design

**FIGURE 1:**
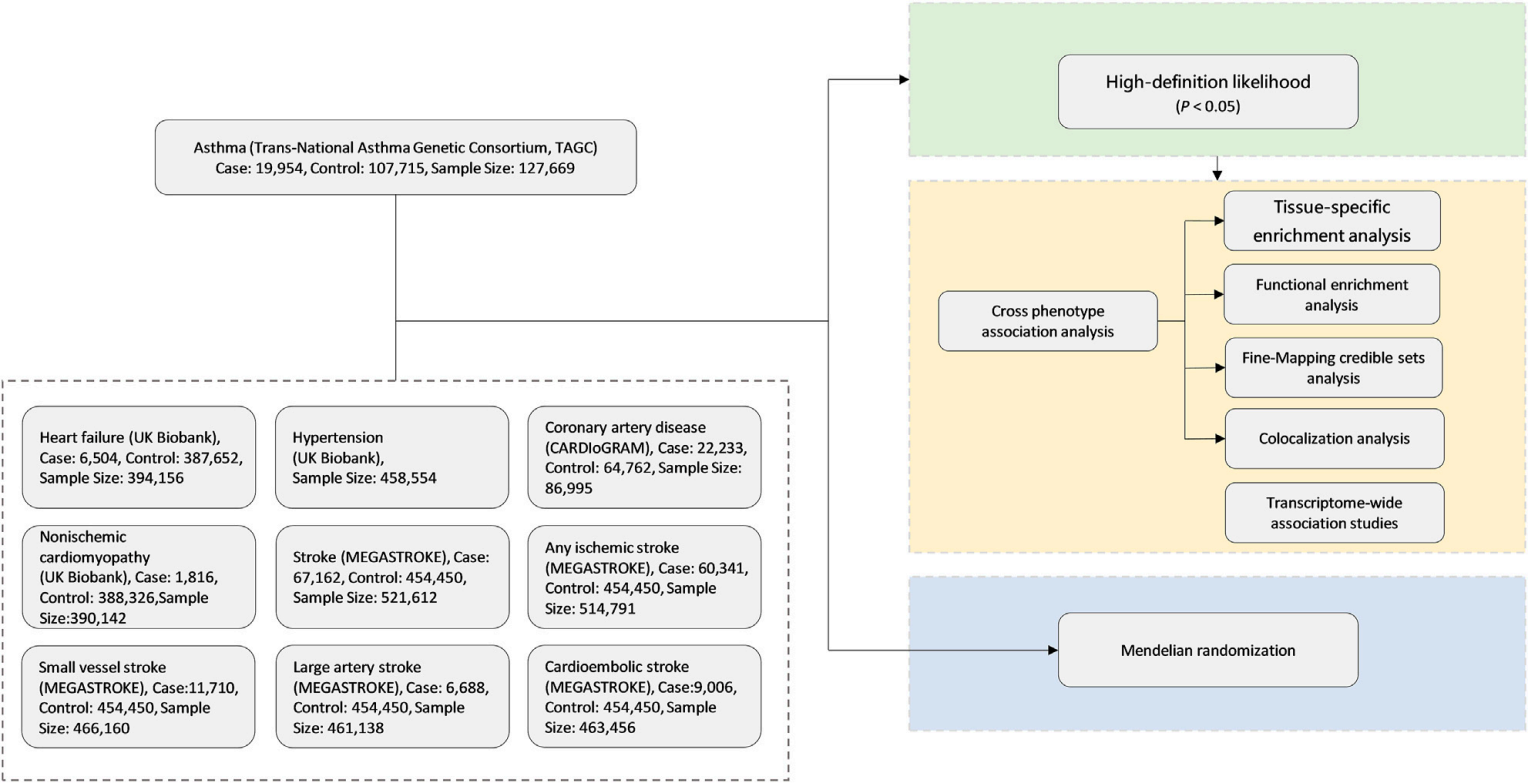
Genome-wide cross-trait analysis study design. CARDIoGRAM, Coronary ARtery Disease Genome-Wide Replication And Meta-Analysis; MEGASTROKE, multi-ancestry genome-wide association study of 520,000 subjects identifies 32 loci associated with stroke and stroke subtypes.

The overall study design is shown in Figure 1. We conducted a large-scale, genome-wide, cross-trait meta-analysis to identify the shared genetic architecture between asthma and CVDs. Our study contains three parts. Firstly, we adopted high-definition likelihood (HDL), a recently developed method, for calculation of genetic correlation between asthma and CVDs to find the significant trait pairs. Secondly, for those significant trait pairs in the first step, we run cross-trait association and meta-analyses to identify the shared loci of significant trait pairs. Finally, we applied MR analyses to evaluate whether asthma is causally associated with CVDs, especially the ones with significant genetic correlations in the first step.

Through literature review and based on the availability of full genome-wide association study (GWAS) data, we chose the following published GWAS for our analyses: 1) asthma (*N* = 127,669), from the Trans-National Asthma Genetic Consortium (TAGC) (Demenais et al., 2018); 2) HF (*N* = 394,156) and non-ischemic cardiomyopathy (NICM) (*N* = 390,142), from the study of Aragam et al. (2018); 3) coronary artery disease (CAD) (*N* = 22,233), from the Coronary ARtery Disease Genome-Wide Replication And Meta-Analysis (CARDIoGRAM) (Schunkert et al., 2011); and 4) all-cause stroke and subtype-specific stroke [any ischemic stroke (AIS), large-artery stroke (LAS), cardioembolic stroke (CES), and small-vessel stroke (SVS)] (*N* = 6,688–67,162), from MEGASTROKE (Malik et al., 2018). The full GWAS summary statistics for all these traits are publicly available for downloading (Supplementary Table S1). All of the datasets were from European populations.

### Genetic Correlation Analysis

HDL was used to estimate the genetic correlation between asthma and CVDs in our genome-wide, cross-trait genetic correlation analysis. HDL used summary data from GWAS to compute the genetic correlation (ranging from −1 to 1), which is similar to linkage disequilibrium score regression (LDSC). However, HDL was reported to reduce the variance of genetic correlation estimates and is therefore more powerful to reveal the potential connections between human complex traits (Ning et al., 2020).

### Cross-Phenotype Association Analyses

GWAS has identified genetic variation for many complex traits, but studies have shown that many detected loci can be associated with multiple traits at the same time. This phenomenon is called cross-phenotype (CP) association, or simply put, pleiotropy (Solovieff et al., 2013). Detection of shared loci for cross-phenotype associations can improve statistical testing and uncover more genetic information between different traits. We used cross-phenotype association analyses (CPASSOC) to identify shared loci between asthma and CVDs in genome-wide scale. We chose CPASSOC over other similar methods/tools because it allows heterogeneity test between traits, controls for population structure and cryptic relatedness, and effectively accounts for sample overlapping. Considering the heterogeneity of different traits in this study, we chose *S*_Het_ as the main statistic. We used the threshold of *P*_SHet_ <5e−8 and *p* < 0.05 to identify CP-associated single-nucleotide polymorphisms (SNPs) that have effects on asthma and each of the CVDs that are included in this study (Zhu et al., 2015). CP-associated SNPs were compared with reported gene and SNPs of asthma and HF with the NHGRI-EBI GWAS catalog (July 2021) in order to test independent replication and find novel loci. Independent loci were extracted after performing SNP prioritization based on linkage disequilibrium for CP-associated SNPs.

### Tissue-Specific Enrichment Analysis

The analyses mentioned above only studied genetics at the DNA level. We next went downstream the chain of the central dogma of molecular biology, to examine tissue-specific gene expression. We first run tissue-specific enrichment analysis (TSEA) to identify gene expressions that are highly enriched or expressed in certain given tissues (Bindea et al., 2009). In our study, we used the gene sets obtained in cross-phenotypic association analysis to calculate tissue-specific gene enrichment and identify tissues that share enriched gene expression between asthma and CVDs. We calculated tissue-specific gene enrichment statistic using the hypergeometric test in the “TissueEnrich” R package (Jain and Tuteja, 2019). The RNA-seq data used in this R package was from the GTEx project (GTEx Consortium, 2015) and Human Protein Atlas (HPA) (Uhlen et al., 2015). In brief, GTEx analyzed RNA sequencing data from a total of 1,641 samples from 43 tissues of 145 individuals to compile a catalog of thousands of tissue-specific and shared regulatory expression quantitative trait loci (eQTL) variants (GTEx Consortium, 2015).

### Functional Enrichment Analysis

Functional enrichment analysis is a method to find a series of over-represented genes or proteins. We used the ClueGO software to assess the enrichment of gene sets in Gene Ontology (GO) biologic process, reactome pathways, and Kyoto Encyclopedia of Genes and Genomes (KEGG) pathways (Bindea et al., 2009). GO annotates genes to biological process, molecular function, and cellular component in a hierarchically structured way (Ashburner et al., 2000). KEGG assigns genes to functional pathways (Kanehisa et al., 2002). The reactome pathway (https://reactome.org) provides molecular details of signal transduction, transport, DNA replication, metabolism, and other cellular processes (Jassal et al., 2020). In this study, we used the gene sets corresponding to significant CP-associated loci obtained in CPASSOC to explore the enrichment and relationship between shared gene sets and GO terms, reactome, and KEGG pathways (Ashburner et al., 2000; Kanehisa et al., 2002; The Gene Ontology Consortium, 2019; Jassal et al., 2020).

### Transcriptome-Wide Association Studies

TWAS enables to evaluate tissue-specific expression–trait associations and the shared expression–trait associations between asthma and CVDs by integrating GWAS summary data and gene expression datasets (Gusev et al., 2016). In our study, we conducted TWAS based on 48 GTEx (version 7) tissue expression weights of the FUSION software package and compared the results with those obtained by TSEA (GTEx Consortium, 2015). We applied Bonferroni correction on TWAS *p*-values, and *P*_Bonferroni_ < 0.05 was considered significant.

### Fine-Mapping Credible Set Analysis

A credible variant set most likely to be causally related in each risk locus can be obtained from fine mapping credible set analysis, replacing the original independent significant loci for subsequent functional studies, with a higher probability to obtain the true causal loci (Schizophrenia Working Group of the Psychiatric Genomics Consortium, 2014; Fachal et al., 2020). In our study, we extracted variants within 500 kb in every locus that were highly linked (*R* ^2^ > 0.4) with CP-associated loci of asthma and CVDs and then used Bayesian likelihood fine-mapping algorithm to determine a 99% plausible set of causal loci, making the identified regions more precise (Wellcome Trust Case Control Consortium et al., 2012).

### Colocalization Analysis

The colocalization analysis is used to assess the colocalization of association loci in two different GWAS by fully Bayesian colocalization analyses, which can help to gain insight into the molecular pathways of complex diseases (Giambartolomei et al., 2014; Hukku et al., 2021). In our study, we used the “coloc.abf” function from the “coloc” R package to conduct genetic colocalization analysis of asthma and CVDs, calculating the probability that each CP-associated locus is a shared genetic causal variant (H4). Loci with posterior probabilities greater than 0.5 were considered colocalized.

### Mendelian Randomization

Mendelian randomization takes advantage of the innate stability and ease of measurement of genetic variation, using it as an instrumental variable (IV) for analysis and thereby obtaining a causal relationship between exposure and outcome (Emdin et al., 2017). Observational studies have found that lung disease is independently associated with CVDs, especially ischemic heart disease and HF (Carter et al., 2019). However, the actual relationship between asthma and CVDs remains unclear. Inverse variance weighting (IVW) was used as the main method to assess causality. Two median-based methods (simple and weighted), MR-Egger and MR-Pleiotropy RESidual Sum and Outlier (MR-PRESSO) methods, were used as sensitivity analyses.

## Results

### Genetic Correlation

Among nine CVDs, we found a significant genetic correlation between asthma and HF (Rg = 0.278, *p* = 0.0005). HDL analyses did not support a significant genetic correlation between asthma and CAD, hypertension (Hp), NICM, stroke, SVS, AIS, CES, and LAS. Estimates of genetic correlation using GWAS summary statistics data are shown in Table 1 (SNP-based heritability estimated by HDL is shown in Supplementary Table S2).

**TABLE 1:** Genetic correlation between asthma and cardiovascular diseases.

Correlation	Rg	Rg SE	*p*
Heart failure	0.278	0.080	0.0005
Coronary artery disease	0.003	0.191	0.9894
Hypertension	0.091	0.059	0.1236
Non-ischemic cardiomyopathy	0.155	0.137	0.2571
Stroke	0.133	0.076	0.0796
Small-vessel stroke	0.156	0.162	0.3347
Any ischemic stroke	0.128	0.076	0.0927
Cardioembolic stroke	0.145	0.086	0.0923
Large-artery stroke	0.253	0.154	0.1008

Rg, genetic correlation estimate; SE, standard error.

### Cross-Phenotype Association Analysis

CPASSOC analyses identified a total of 145 independent loci (*P*_SHet_ <5 × 10^−8^ and trait-specific *p* < 0.05), which maps to 99 genes (Supplementary Table S3). We compared the reported gene and SNPs of asthma and HF with the NHGRI-EBI GWAS catalog (July 2021) and found that 15 of 145 SNPs were novel associations not previously reported for either asthma or HF (Supplementary Table S4). Independent loci were extracted after performing SNP prioritization based on linkage disequilibrium (LD) for CP-associated SNPs and are shown in Table 2. The threshold *P* of LD is 5e−5, with clump results using a distance of 500 kb and using an *R* ^2^ of 0.4. The strongest association signal was observed near the *GSDMB* gene on chromosome 17 (*P*_SHet_ = 7.13 × 10^−44^). We also observed the association signal near *ORMLD3* (*P*_SHet_ = 1.82 × 10^−43^) as well as *IKZF3* (*P*_SHet_ = 1.07 × 10^−41^) on chromosome 17. Previous studies reported that *GSDMB* and *ORMDL3* are associated with asthma (Zhao et al., 2015), but their association with HF was not reported. *IKZF3* is associated with the IL-2 signaling pathway and NF-κB signaling pathway, which are involved in regulating *BCL2* expression and controlling apoptosis (Kioussis, 2007). Our HDL findings suggest that the overall strong positive genetic correlation between asthma and HF is driven by these identified shared genetic loci.

**TABLE 2:** Summary of independent loci associated with asthma and heart failure in cross-phenotype association analysis.

SNP	CHR	POS	Alt. allele	Ref. allele	Asthma *P*	HF *P*	Meta-analysis *P*	DIST	Nearest gene to the loci
rs2305479	17	38062217	C	T	1.00E−42	3.76E−04	7.13E−44	0	*GSDMB*
rs4795408	17	38107627	G	A	2.12E−36	2.18E−04	3.12E−37	6.64	*LRRC3C*
rs7207600	17	38091660	G	A	4.72E−29	8.34E−03	1.58E−29	6.066	*LRRC3C*
rs2952156	17	37876835	A	G	7.55E−29	2.14E−02	2.60E−29	0	*ERBB2*
rs3859192	17	38128648	C	T	5.38E−28	1.92E−02	2.03E−28	0	*GSDMA*
rs11078930	17	38141955	C	T	1.49E−20	3.67E−02	1.24E−20	0	*PSMD3*
rs876493	17	37824545	G	A	1.92E−16	1.07E−02	2.42E−16	0	*PNMT*
rs8069451	17	37504933	T	C	1.50E−08	4.67E−02	3.70E−08	0	*FBXL20*
rs2158177	5	131984058	A	G	6.18E−11	2.32E−02	1.28E−10	3.745	*RAD50*
rs2248116	5	131804347	C	A	6.67E−11	3.29E−02	1.38E−10	0	*C5orf56*
rs12521097	5	131575338	G	A	1.70E−08	1.91E−02	4.22E−08	11.782	*P4HA2*
rs10124250	9	6161686	C	T	2.40E−12	3.70E−02	4.41E−12	54.099	*IL33*
rs1333042	9	22103813	A	G	2.97E−04	3.23E−07	2.53E−09	0	*CDKN2B-AS1*
rs2383206	9	22115026	A	G	4.05E−04	4.95E−07	4.95E−09	0	*CDKN2B-AS1*
rs10811650	9	22067593	A	G	1.15E−03	1.68E−06	3.74E−08	0	*CDKN2B-AS1*

SNP, single-nucleotide polymorphism; CHR, chromosome; POS, position of the clumping region on the chromosome; Alt. allele, alternative allele; Ref. allele, reference allele; HF, heart failure; DIST: the smallest (in absolute value) signed distance of the nearest gene to the loci. The threshold *P* of linkage disequilibrium is 5e−5. Clump results use a distance of 500 kb and *R* ^2^ of 0.4. SNP positions are reported according to Build GRch37.

### Tissue-Specific Enrichment Analysis

**FIGURE 2:**
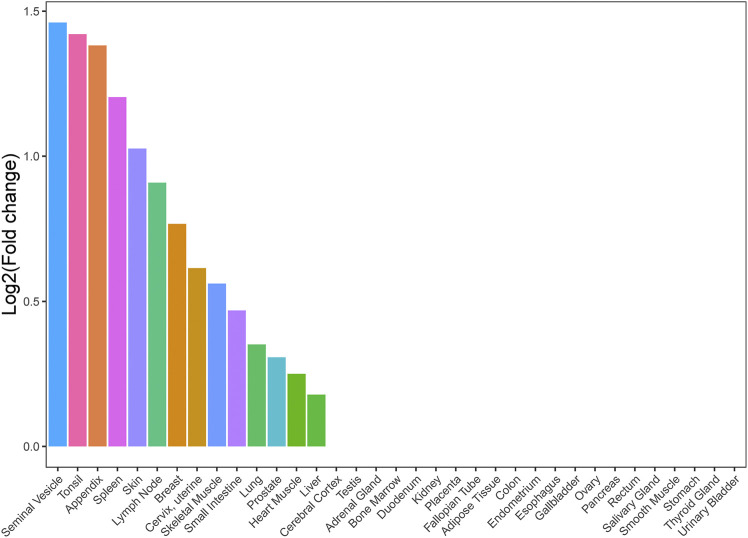
Tissue enrichment analysis result of shared genes between asthma and HF. Vertical axis: the logarithm of tissue expression enrichment fold change based on two; horizontal axis: 35 independent tissues. HF, heart failure.

We conducted a TSEA for asthma and HF with significant genetic correlation using GTEx pilot data to assess whether shared genes between the two traits are enriched for expression in tissues. We chose all tissues in GTEx in order to provide complete analysis results and explore some potential tissues associated with shared genes between asthma and HF. We found that shared genes between asthma and HF were significantly enriched in 14 tissues, including the seminal vesicle, tonsil, appendix, spleen, skin, lymph nodes, breast, cervix and uterus, skeletal muscle, small intestine, lung, prostate, cardiac muscle, and liver (Figure 2). These results support that there is a suite of complex biological process underlying the shared genetic architecture between asthma and HF. It is not driven by a single tissue type or a single biological process such as inflammation.

### Functional Enrichment Analysis

In order to understand the biological processes between asthma and HF, we conducted an analysis of GO biologic process, KEGG pathways, and reactome pathways (false discovery rate <0.05). The GO analysis indicated that shared genes between asthma and HF were significantly enriched in nuclear receptor activity, mating behavior, cyclin-dependent protein serine/threonine kinase regulator activity, granulocyte differentiation, regulation of B cell proliferation, negative regulation of lymphocyte differentiation, regulation of transcription initiation from RNA polymerase II promoter, and regulation of leukocyte apoptotic process (Supplementary Figure S1; Supplementary Table S5). To further know the functional pathways, KEGG pathway analyses indicated that shared genes were significantly enriched in hematopoietic cell lineage, Fc epsilon RI signaling pathway, and acute myeloid leukemia (Supplementary Table S6). In additional analyses of reactome pathways, we found that shared association signals for asthma and HF were significantly enriched in the nuclear receptor transcription pathway and interleukin receptor SHC signaling (Supplementary Table S7).

### Transcriptome-Wide Association Studies

TWAS analyses were used to evaluate tissue-specific expression–trait associations and the shared expression–trait associations between asthma and HF, to identify shared genes revealing the shared genetic architecture or mechanisms. We compared the asthma and HF reported gene with the NHGRI-EBI GWAS catalog in July 2021, respectively, and found that 829 of 1,619 asthma genes were novel to asthma and 199 of 254 HF genes were novel to HF (significant genes were identified by *P*_Bonferroni_ <0.05). TWAS results of asthma and HF are shown in Supplementary Tables S8 and S9.

A large number of TWAS-significant associations for asthma were identified in tissues that are part of the nervous system, digestive system, exo-/endocrine system, as well as cardiovascular system. The strongest expression–trait associations for asthma were observed at *PNMT* (phenylethanolamine N-methyltransferase) on chromosome 17 (the strongest association at the adipose visceral omentum, *P*_TWAS_ = 6.58 × 10^−46^), which catalyzes the synthesis of epinephrine from norepinephrine (Mahmoodi et al., 2020). In addition, *GSDMB* (the strongest association at the small intestine terminal ileum, *P*_TWAS_ = 1.37 × 10^−44^), *RP11-387H17.4* (the strongest association at the coronary artery, *P*_TWAS_ = 1.43 × 10^−43^), *GSDMB* (the strongest association at transverse colon, *P*_TWAS_ = 1.00 × 10^−42^), and *ORMDL3* (the strongest association at cell-transformed fibroblasts, *P*_TWAS_ = 7.81 × 10^−43^) were also TWAS significant (Supplementary Figure S2). These results were consistent with those reported by Demenais et al. (2018), which studied asthma risk loci that colocalize with immune cell enhancer marks.

A large number of TWAS-significant associations for HF were identified in tissues that are part of the digestive system, nervous system, exo-/endocrine system, and cardiovascular system, similar with asthma TWAS. The strongest expression–trait associations for asthma were observed at *RP11-378J18.8* on chromosome 1 (the strongest association at the uterus, *P*_TWAS_ = 1.43 × 10^−7^). In addition, *MAP3K7CL* (the strongest association at cell-transformed fibroblasts, *P*_TWAS_ = 4.86 × 10^−7^), *LINC00189* (the strongest association at the spleen, *P*_TWAS_ = 1.25 × 10^−6^), and *MRPL35* (strongest association at coronary artery, *P*_TWAS_ = 1.66 × 10^−6^; the strongest association at the sigmoid colon, *P*_TWAS_ = 1.51 × 10^−6^) were also TWAS significant (Supplementary Figure S3).

**FIGURE 3:**
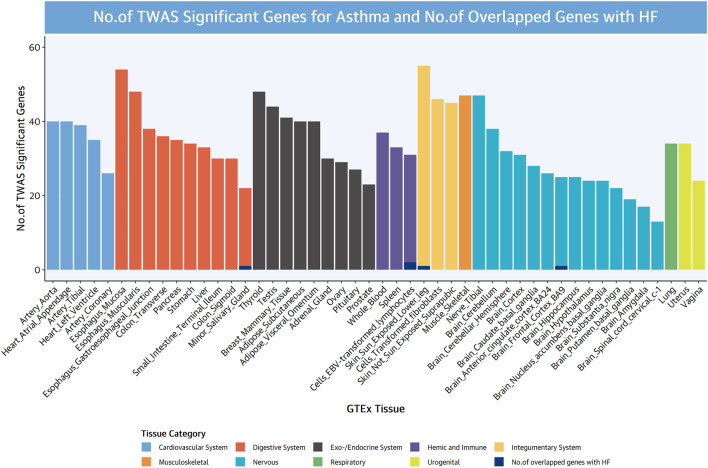
Numbers of significant genes for asthma and numbers of overlapped genes with HF. Significant genes were identified by *P*_Bonferroni_ <0.05.

We examined TWAS genes shared between asthma and HF and identified five TWAS-significant genes shared between asthma and HF, mainly from the hemic and immune systems (two TWAS-significant genes at EBV-transformed lymphocytes), digestive system (one TWAS-significant gene at the minor salivary gland), integumentary system (one TWAS-significant at the skin), and nervous system (one TWAS-significant at the brain frontal cortex) (Figure 3).

We identified three genes (*GSDMA* in chr17, *GSDMB* in chr17, and *ORMDL3* in chr17) with statistically independent genetic effects from all of the shared TWAS genes between asthma and HF (Table 3). *GSDMA* and *GSDMB* induce inflammatory response and cell pyroptosis (Ding et al., 2016). *ORMLD3* could facilitate the survival of splenic B cells *via* an ATF6α-endoplasmic reticulum stress-beclin1 autophagy regulatory pathway (Dang et al., 2017).

**TABLE 3:** TWAS significant genes shared between asthma and HF.

Gene	HF				Asthma			
	BEST. GWAS.ID	TWAS.Z	*P*_Bonferroni_	Tissue	BEST. GWAS.ID	TWAS.Z	*P*_Bonferroni_	Tissue
*GSDMA*	rs11557467	−4.0641	0.0283	Sun-exposed lower leg skin	rs2305479	−12.9856 to 10.9691	1.12E−35 to 2.60E−19	Adipose subcutaneous, adipose visceral omentum, adrenal gland, aortic artery, coronary artery, tibial artery, brain substantia nigra, breast mammary tissue, cell-transformed fibroblasts, sigmoid colon, transverse colon, gastroesophageal junction of the esophagus, esophageal mucosa, esophagus muscularis, heart atrial appendage, heart left ventricle, lung, minor salivary gland, skeletal muscle, tibial nerve, pancreas, prostate, sun-exposed lower leg skin, small intestine terminal ileum, spleen, stomach, testis, and thyroid
*GSDMB*	rs11557467	3.7006 to 3.7704	0.0209 to 0.0441	Minor salivary gland, cells EBV-transformed lymphocytes	rs2305479	−8 to 14.0094	3.96E−42 to 9.58E−09	Adipose subcutaneous, adrenal gland, tibial artery, brain cortex, brain nucleus accumbens basal ganglia, cells EBV-transformed lymphocytes, cell-transformed fibroblasts, transverse colon, esophageal mucosa, esophagus muscularis, lung, minor salivary gland, tibial nerve, ovary, pancreas, sun-exposed lower leg skin, small intestine terminal ileum, spleen, stomach, and whole blood
*ORMDL3*	rs11557467	3.7006 to 3.8588	0.0247 to 0.0441	Brain frontal cortex BA9, cells EBV-transformed lymphocytes	rs2305479	−12.7 to 13.7191	5.08E−40 to 9.07E−06	Adipose subcutaneous, adrenal gland, aortic artery, brain frontal cortex BA9, breast mammary tissue, cells EBV-transformed lymphocytes, cell-transformed fibroblasts, sigmoid colon, esophageal mucosa, esophagus muscularis, lung, ovary, suprapubic skin not sun exposed, sun-exposed lower leg skin, spleen, stomach, thyroid, and whole blood

CHR, chromosome; HF, heart failure; TWAS, transcriptome-wide association scan.

### Fine-Mapping and Colocalization Analyses

To more accurately assess whether asthma and HF share common genetic variants within a given region, we conducted a fine-mapping analysis. The credible set of SNPs for each shared locus is shown in the Supplementary Material (Supplementary Table S10). The colocalization analysis showed that 22 out of 145 loci (H4 > 0.5) share causal variants between asthma and HF (Supplementary Table S11).

### Mendelian Randomization

**FIGURE 4:**
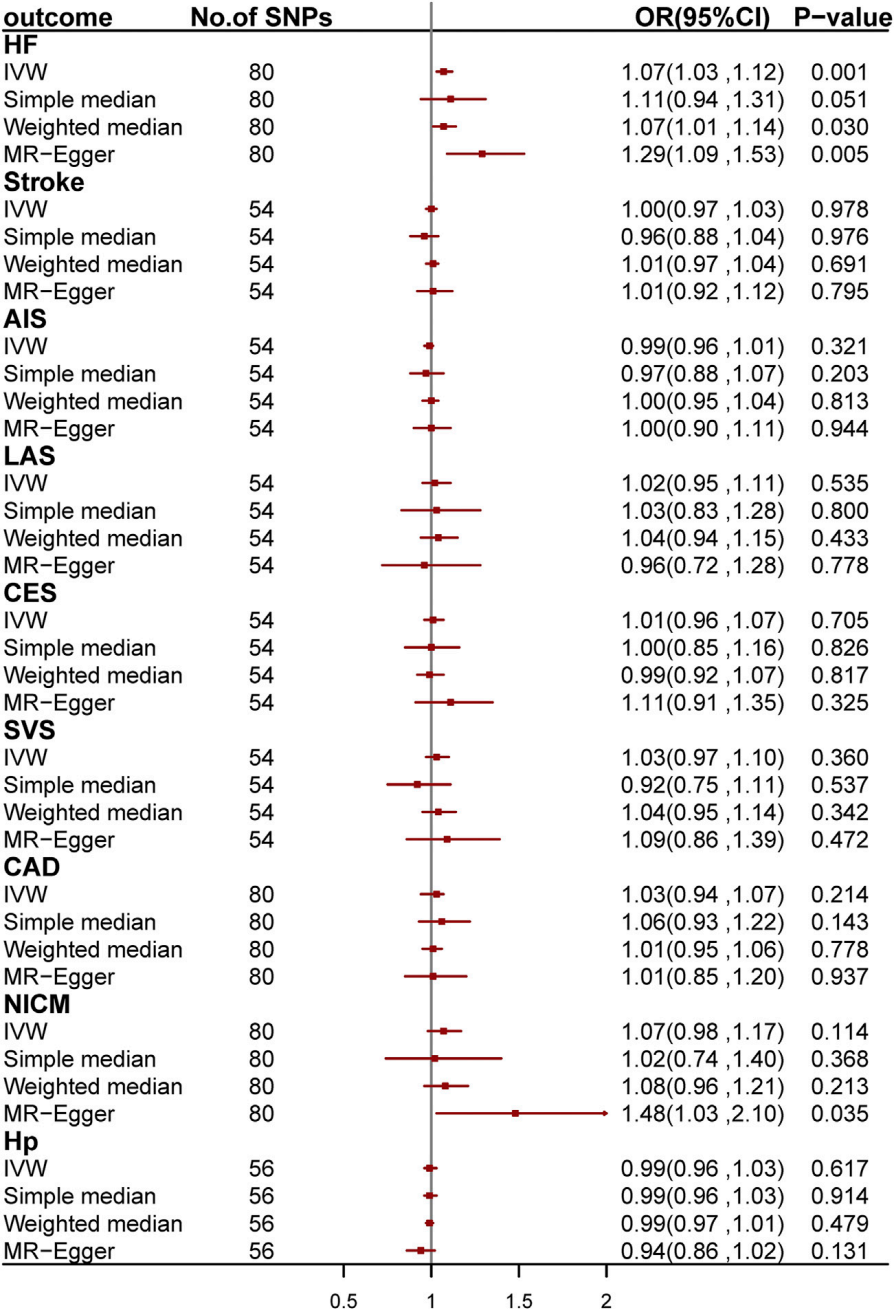
Odds ratio for the association among genetically predicted asthma and cardiovascular-related traits. OR, odds ratio; 95%CI, 95% confidence interval; AIS, any ischemic stroke; CES, cardioembolic stroke; LAS, large-artery stroke; SVS, small-vessel stroke; CAD, coronary artery disease; HF, heart failure; NICM, non-ischemic cardiomyopathy; Hp, hypertension.

We conducted two-sample MR analyses to investigate the causal relationship between asthma and CVDs by using 54, 80, 80, 80, and 56 genetic variants as IV for stroke and its subgroups, CAD, HF, NICM, and Hp, respectively (Figure 4; Supplementary Tables S11–S21). After Bonferroni correction, we found a causal relationship between asthma and HF. The odds ratio (OR) is 1.07 [95% confidence interval (CI): 1.03–1.12; *p* = 1.31 × 10^−3^] per one-unit increase in log_e_ odds of asthma. We further validated the causal effect by applying MR-PRESSO regression, which is robust to bias due to horizontal pleiotropy (Bowden et al., 2015; Burgess and Thompson, 2017).

## Discussion

We used large-scale GWAS summary statistics to analyze the shared genetic architecture and causal associations between asthma and CVDs. First, we found a strong positive genetic correlation and further identified 145 shared significant loci between asthma and HF, of which 15 were novel. Second, we found that these shared loci were significantly enriched in 14 tissues and B-cell proliferation, Fc epsilon RI signaling, and nuclear receptor transcription pathway using functional analysis. Third, we found that shared genes between asthma and HF are focused on the hemic and immune systems, digestive system, integumentary system, and nervous system using TWAS analysis. Finally, we found a causal relationship between asthma and HF using MR analysis.

A previous study showed that the risks of HF were increased in asthma, COPD, and ACOS (Ingebrigtsen et al., 2020). In the present study, we have identified 145 shared loci between asthma and HF through large-scale, genome-wide, cross-trait meta-analysis, of which 15 were novel associations, and all the 145 shared loci did not achieve genome-wide trait-specific significance for both asthma and HF GWAS summary statistics, which demonstrated the great statistical power of our cross-trait meta-analysis.

The top locus common to the asthma and HF was rs2305479 near *GSDMB*. *GSDMB* is an important member of the *GSDMS* family, like *GSDMA* confirmed in TWAS, whose most important function is to induce inflammatory response and cell pyroptosis (Ding et al., 2016). Therefore, *GSDMB* may affect myocardial cell survival through its involvement in the process of pyroptosis. In addition, we found that asthma was causally related with HF (per one-unit increase in log OR of asthma). The result was consistent with a large cohort study (*n* = 203,595), which found that asthma was associated with a 2.14-fold (95% CI: 2.06, 2.22) hazard of HF (Iribarren et al., 2012). Previous studies have demonstrated that inflammatory processes are likely involved in the pathophysiology of asthma, atherosclerosis, and endothelial dysfunction (Fenta et al., 2010; Yun et al., 2012). A previous study indicates that cytokines that are up-regulated in asthma may promote the migration and activation of inflammatory cells implicated in atherogenesis (Halasz et al., 2002; Zietkowski et al., 2007; Zietkowski et al., 2010), and cytokines are also important mediators of bronchoconstriction in asthma (Hiemstra et al., 2015). Leukotrienes as a kind of cytokines found in asthmatic bronchioles also exhibits strong proinflammatory activities in cardiovascular tissues (Spanbroek et al., 2003; Gurgone et al., 2020). Previous studies have shown that pyroptosis not only plays an important role in infectious diseases but is also associated with CVDs, central nervous system diseases, and tumors (Gong et al., 2020; Zhou et al., 2020). TSEA showed that shared genes were enriched in 14 tissues, including the seminal vesicle, tonsil, appendix, spleen, skin, lymph nodes, breast, cervix and uterus, skeletal muscle, small intestine, lung, prostate, cardiac muscle, and liver. Prostaglandins related to the seminal vesicle are suggested to regulate allergic diseases (Peebles, 2019). Prostaglandins and leukotrienes are involved in numerous homeostatic biological functions and inflammation (Funk, 2001). Our TWAS reported tissue enrichment from the hemic and immune systems, digestive system, integumentary system, and nervous system. Another top association for asthma and HF was *ORMDL3*, which could facilitate the survival of splenic B cells *via* an ATF6α-endoplasmic reticulum stress-beclin1 autophagy regulatory pathway (Dang et al., 2017). *GSDMB* and *ORMDL3* were also confirmed to be shared between asthma and HF using TWAS in our study. In addition, *IKZF3* was confirmed in CPASSOC to be associated with the IL-2 signaling pathway and NF-κB signaling pathway, which are involved in regulating *BCL2* expression and controlling apoptosis (Kioussis, 2007).

The functional analysis provided biologic insights into the shared genes between asthma and HF, and nuclear receptor-related pathway was found significant in GO and reactome pathway. The nuclear receptor superfamily includes retinoic acid receptors (RAR) and retinoid X receptors (RXR), whose effects on cell growth and survival can be modulated therapeutically by small-molecule ligands (de Lera et al., 2007). All-trans retinoic acid (ATRA) is an active metabolite of vitamin A, which is associated with increased *ORMDL3* and shows protective effects on asthma, including maintaining airway epithelial integrity, inhibiting asthma effector cell differentiation, modulating immune response, etc. (Day et al., 2006; Zhuang et al., 2013; Garcia-Sanchez et al., 2016). Some studies have reported that retinoic acid (RA) is important for the development of the heart (Pan and Baker, 2007; Olsen and Blomhoff, 2020). Experimental studies showed that RA treatment could improve cardiac injury of gestational diabetes mellitus mice (Liu et al., 2020).

This study has several strengths. This is the first analysis to identify the shared genetic architecture of asthma and HF using the large GWAS data. HDL that was used to explore genetic correlations in this study and was able to reduce the genetic correlation variance by 60%, equivalent to a 2.5-fold increase in sample size (Ning et al., 2020). We used CPASSOC to better understand the shared genetic architecture between asthma and HF. CPASSOC allows for heterogeneity between traits, controls population structure and invisible relatedness, and allows for sample overlap. We found the seminal vesicle to be one of the major tissues and the hemic and immune systems to be one of the major systems that were significant between asthma and HF. These results contribute to further understanding of the association of asthma and HF and provide additional suggestions for possible shared mechanisms.

This study has some limitations. First, the data used in the present study was limited by diseases diagnosis, medication administration, and so on. However, GWAS summary statistics conducted study-specific quality control to ensure the quality. Second, the summary statistics limit us to segregate asthma patients by disease onset (childhood vs. adulthood), and lung function test data that reflect asthma severity are missing. Further studies are required to explore the genetic association of different asthma types with CVD risk. Third, our study is only limited to assessing the shared genetic architecture between asthma and CVDs, but environmental factors are different for specific traits. Therefore, a gene–environment interaction study between asthma and CVDs is encouraged. Finally, our study was limited to European ancestry, and shared genetic architecture in other ethnic groups are uncertain.

## Conclusion

In summary, our findings provide strong evidence of genetic correlations and causal association between asthma and HF, which support that early preventive measures of CVDs can be taken in asthmatic patients. These results provide a novel insight of the shared genetic basis between asthma and HF from molecular and functional levels and contribute to further laboratory investigation and clinical studies.

## Data Availability

The original contributions presented in the study are included in the article/Supplementary Material, further inquiries can be directed to the corresponding author.
