# Supplementary figures and images for: Shared Genetic Architecture and Causal Relationship Between Asthma and Cardiovascular Diseases: A Large-Scale Cross-Trait Analysis
Source: Front Genet. 2022 Jan 20;12:775591. doi: 10.3389/fgene.2021.775591 (PMC8811262; doi:10.3389/fgene.2021.775591)

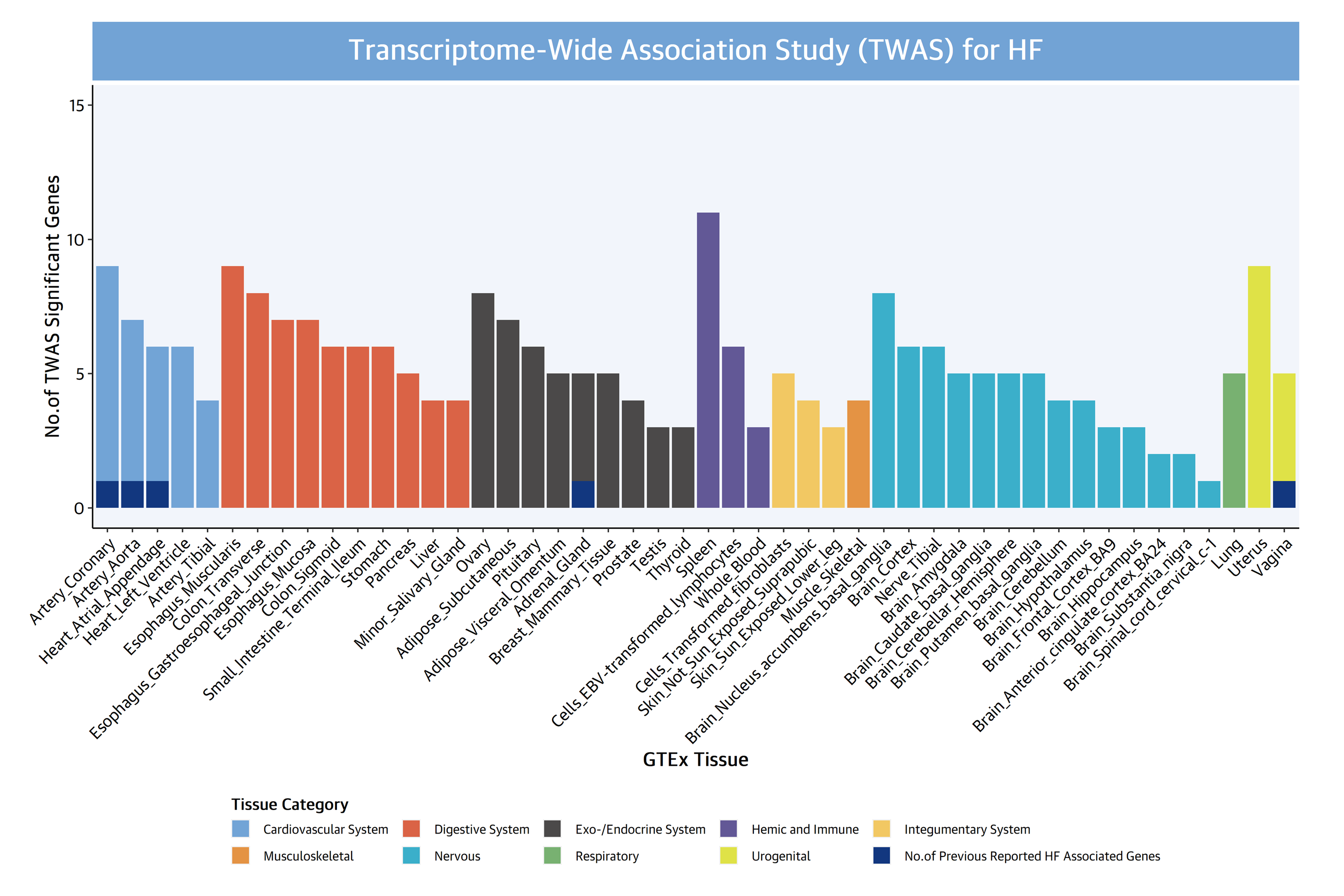

Supplement: Supplementary file 1 [file Image3.TIF]

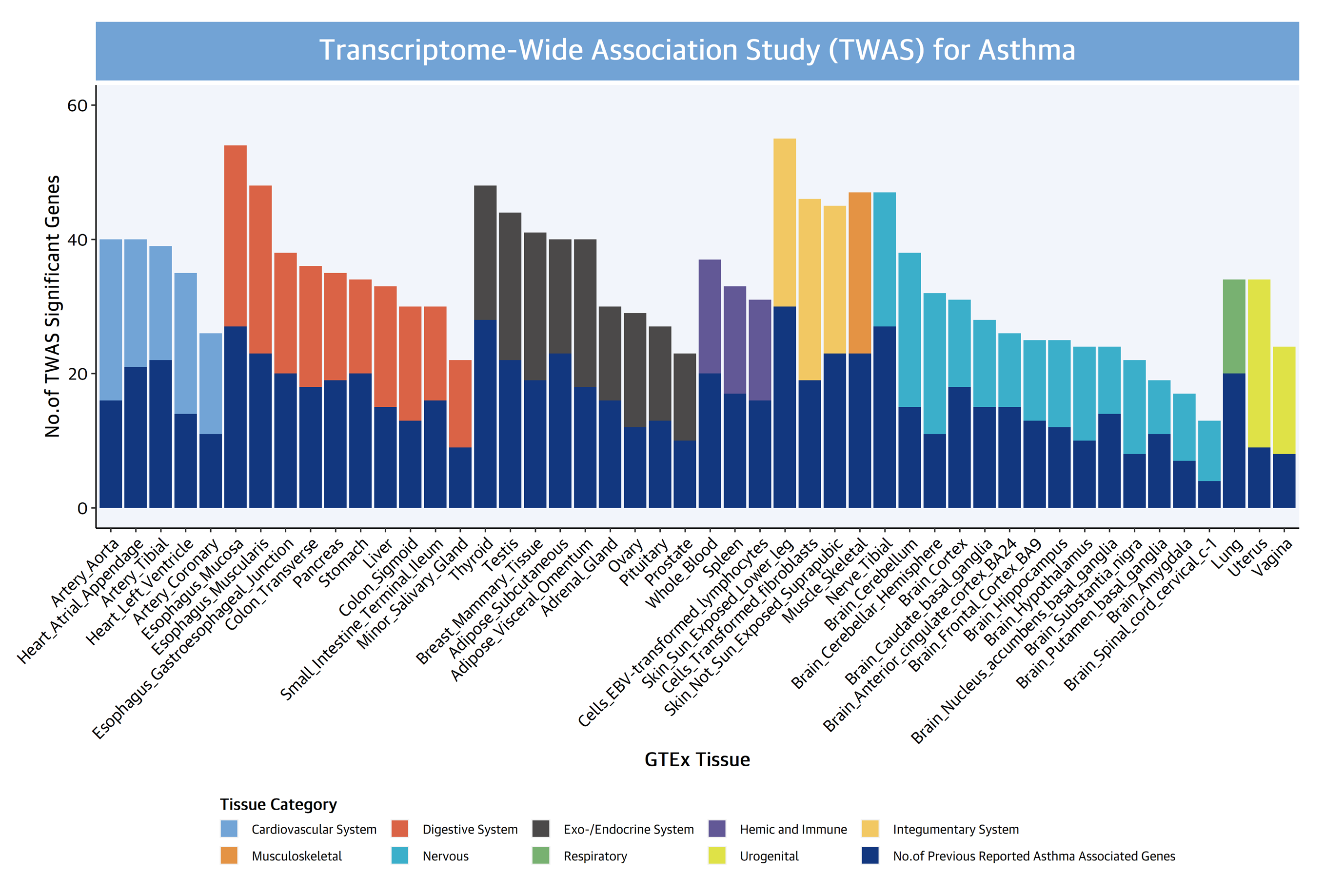

Supplement: Supplementary file 2 [file Image2.TIF]

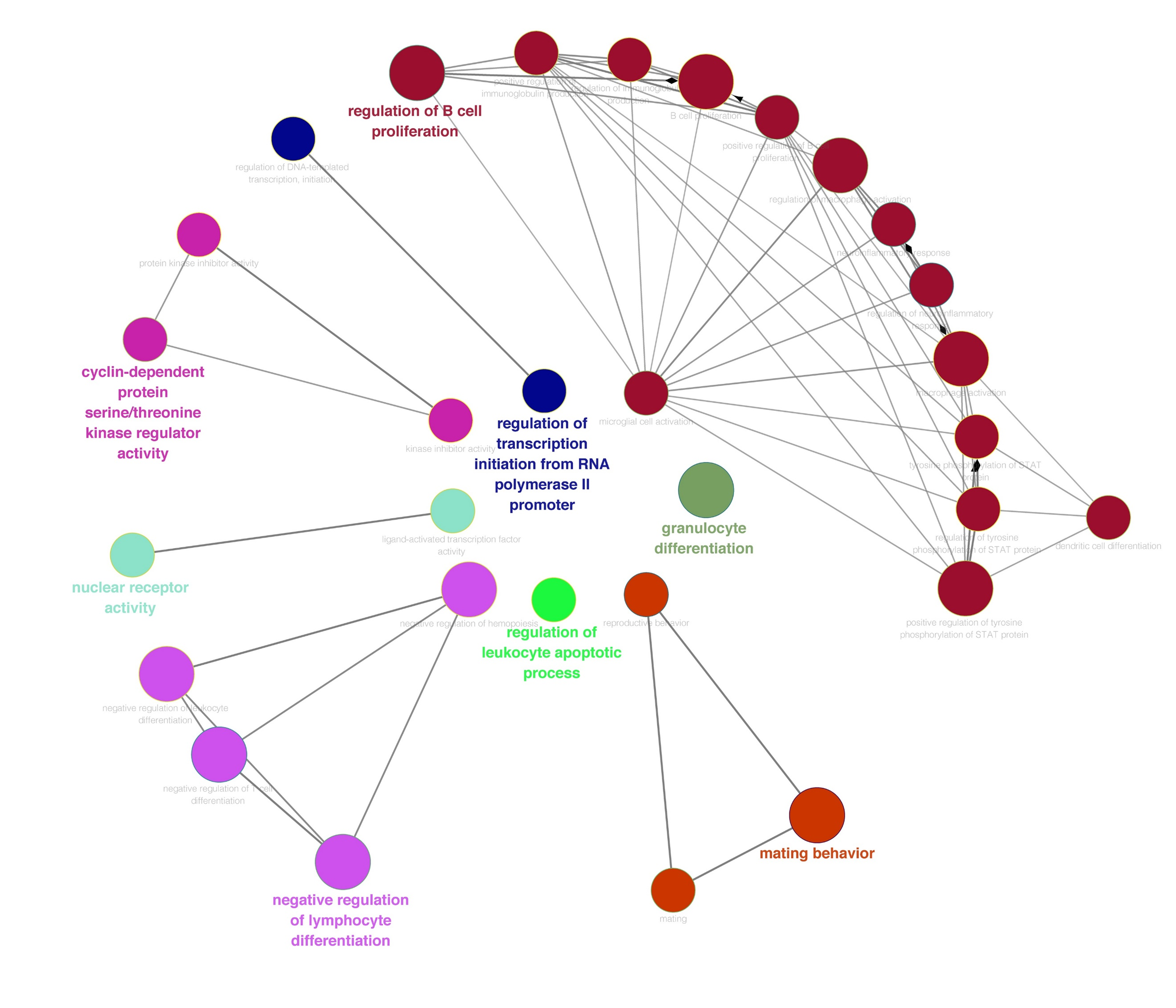

Supplement: Supplementary file 3 [file Image1.TIF]
